# Audiogram Estimation Performance Using Auditory Evoked Potentials and Gaussian Processes

**DOI:** 10.1097/AUD.0000000000001570

**Published:** 2024-09-12

**Authors:** Michael Alexander Chesnaye, David Martin Simpson, Josef Schlittenlacher, Søren Laugesen, Steven Lewis Bell

**Affiliations:** 1 National Acoustic Laboratories, Hearing Australia, Sydney, Australia; 2 Faculty of Engineering and the Environment, Institute of Sound and Vibration Research, University of Southampton, Southampton, United Kingdom; 3 Division of Psychology and Language Sciences, University College London, London, United Kingdom; 4 Interacoustics Research Unit, C/O Technical University of Denmark, Lyngby, Denmark.

**Keywords:** Active learning, Audiogram estimation, Auditory brainstem responses, Gaussian processes

## Abstract

**Objectives:**

Auditory evoked potentials (AEPs) play an important role in evaluating hearing in infants and others who are unable to participate reliably in behavioral testing. Discriminating the AEP from the much larger background activity, however, can be challenging and time-consuming, especially when several AEP measurements are needed, as is the case for audiogram estimation. This task is usually entrusted to clinicians, who visually inspect the AEP waveforms to determine if a response is present or absent. The drawback is that this introduces a subjective element to the test, compromising quality control of the examination. Various objective methods have therefore been developed to aid clinicians with response detection. In recent work, the authors introduced Gaussian processes (GPs) with active learning for hearing threshold estimation using auditory brainstem responses (ABRs). The GP is attractive for this task, as it can exploit the correlation structure underlying AEP waveforms across different stimulus levels and frequencies, which is often overlooked by conventional detection methods. GPs with active learning previously proved effective for ABR hearing threshold estimation in simulations, but have not yet been evaluated for audiogram estimation in subject data. The present work evaluates GPs with active learning for ABR audiogram estimation in a sample of normal-hearing and hearing-impaired adults. This involves introducing an additional dimension to the GP (i.e., stimulus frequency) along with real-time implementations and active learning rules for automated stimulus selection.

**Methods:**

The GP’s accuracy was evaluated using the “hearing threshold estimation error,” defined as the difference between the GP-estimated hearing threshold and the behavioral hearing threshold to the same stimuli. Test time was evaluated using the number of preprocessed and artifact-free epochs (i.e., the sample size) required for locating hearing threshold at each frequency. Comparisons were drawn with visual inspection by examiners who followed strict guidelines provided by the British Society of Audiology. Twenty-two normal hearing and nine hearing-impaired adults were tested (one ear per subject). For each subject, the audiogram was estimated three times: once using the GP approach, once using visual inspection by examiners, and once using a standard behavioral hearing test.

**Results:**

The GP’s median estimation error was approximately 0 dB hearing level (dB HL), demonstrating an unbiased test performance relative to the behavioral hearing thresholds. The GP additionally reduced test time by approximately 50% relative to the examiners. The hearing thresholds estimated by the examiners were 5 to 15 dB HL higher than the behavioral thresholds, which was consistent with the literature. Further testing is still needed to determine the extent to which these results generalize to the clinic.

**Conclusions:**

GPs with active learning enable automatic, real-time ABR audiogram estimation with relatively low test time and high accuracy. The GP could be used to automate ABR audiogram estimation or to guide clinicians with this task, who may choose to override the GP’s decisions if deemed necessary. Results suggest that GPs hold potential for next-generation ABR hearing threshold and audiogram-seeking devices.

## INTRODUCTION

The audiogram shows a subject’s hearing threshold as a function of frequency, and is routinely used in the clinic to specify hearing loss characteristics and fit hearing aids (British Society of Audiology 2021). Usually, the audiogram can be estimated through pure-tone audiometry, that is, a behavioral hearing test that relies on voluntary responses to locate hearing thresholds. However, this method is not always applicable, as some subjects, such as newborns and some adults with cognitive impairments, may be unable to provide reliable behavioral responses. In these cases, the audiogram can be estimated using objective measures of hearing that do not rely on voluntary responses.

A commonly used objective measure of hearing is the auditory brainstem response (ABR), which represents a brief change in brain activity triggered by an acoustic stimulus (Hall 2006). The ABR can be measured noninvasively using scalp electrodes, and comprises a series of peak and trough voltage amplitudes, known as “Jewett waves” (Jewett et al. 1970; Hall 2006). The challenge is that the ABR is hidden in the background activity, which can be an order of magnitude larger than the ABR. To reliably detect the ABR, it is common practice to present many stimuli to the subject, and average the short time intervals following stimulus onset to reduce “noise.” The cost, however, is relatively long test times, especially when multiple ABR measurements are needed. For example, mean test times for estimating eight hearing thresholds (four frequencies per ear) in newborns previously ranged from 30 to 60 min (Janssen et al. 2010; Sininger et al. 2018). Moreover, when testing newborns, the time window for data collection tends to be limited to when infants are asleep to reduce movement artifacts. If the infant wakes, testing may need to be stopped, potentially resulting in additional appointments to finalize the test (British Society of Audiology 2021). Efficient methods for quickly evaluating hearing ability within these limited time windows are thus desirable, especially in busy clinics where short test times are critical.

Besides long test times, determining the presence or absence of an ABR can be a challenging task. This is usually entrusted to highly trained clinicians who visually inspect the averaged waveforms (British Society of Audiology 2019). Although potentially quite sensitive, visual inspection outcomes are known to vary within and between examiners (Vidler & Parkert 2004; Zaitoun et al. 2016), which thus introduces a subjective, error-prone element to the procedure. To reduce subjectivity, and improve test accuracy and efficiency, numerous statistical approaches have been developed to assist clinicians with ABR hearing tests (see also Discussion). For the present work, the main focus was on a recently developed technique for objective ABR hearing threshold and/or audiogram estimation, and involves Gaussian processes (GPs) in combination with active learning rules (Chesnaye et al. 2023). The GP is a Bayesian approach for nonlinear regression (Rasmussen & Williams 2006), which was previously used to estimate the ABR’s amplitude-intensity growth function, that is, ABR amplitude across stimulus levels. Active learning rules were also designed to automatically adjust the stimulus and efficiently locate hearing threshold. For an accessible, beginner-friendly starting point to GPs with active learning, see also Gramacy (2021).

The GP is attractive for ABR hearing threshold estimation, first because it no longer utilizes repeated null hypothesis significance testing. The latter is used by most existing methods, which aim to infer hearing threshold from a series of sequentially applied statistical tests that evaluate the null hypothesis of “ABR absent” (Özdamar et al. 1990; Bogaerts et al. 2009; Berninger et al. 2014; Wang et al. 2021). The drawback is that repeated hypothesis testing inflates the false-positive rate (FPR; Armitage et al. 1969), and complex sequential test strategies are needed to maintain control over the test’s significance level (Stürzebecher et al. 2005; Chesnaye et al. 2020; Zanotelli et al. 2020). The GP approach, however, adopts a Bayesian framework for parameter estimation, thus circumventing the need for complex sequential test strategies. This ultimately helps to simplify the procedure while also providing greater flexibility in terms of how long and how often data can be analyzed.

The GP is also attractive because it can learn and exploit the correlation structure underlying the ABR waveforms. It is well-known that ABR measurements are correlated across stimulus levels and frequencies (Picton 2011), but this is typically overlooked by most detection methods. These correlations hold valuable information, and are routinely exploited by clinicians: ABR estimates at high-level measurements, for example, may be used to inform the likely presence of an ABR at lower levels. It would be beneficial for the objective detection method to also consider these correlations, not only to obtain a more powerful test, but also to ensure that the method’s output is aligned with the clinician’s intuitions and expertise, as this may lead to a more predictable and trustworthy detector. It is perhaps also worth mentioning that GP’s have previously been used in the related field of behavioral audiogram estimation (Song et al. 2015; Schlittenlacher et al. 2018).

In previous work, GPs with active learning proved effective for ABR hearing threshold estimation in simulated data where it reduced test time by approximately 50% relative to a sequentially applied Hotelling *T*^2^ test (Chesnaye et al. 2023). The main goal for the present work was to adapt the GP approach for ABR audiogram estimation, and to evaluate its performance in a cohort of normal-hearing (NH) and hearing-impaired (HI) adults. This involves introducing an additional dimension to the GP (i.e., stimulus frequency) along with efficient implementations for real-time data analysis and active learning rules for automated stimulus selection. To establish a benchmark to compare against, subject audiograms were also estimated using conventional visual inspection by examiners, who followed strict guidelines provided by a modified British Society of Audiology (BSA) protocol (British Society of Audiology 2019; see also Visual inspection by examiners).

## MATERIALS AND METHODS

This section describes the subject-recorded ABR data for the assessment (see NH and HI ABR data), along with GPs with active learning (see ABR audiogram estimation using GPs and active learning) and visual inspection by clinicians (see Visual inspection by examiners) for audiogram estimation.

### NH and HI ABR Data

Ethical approval was granted by the Faculty Ethics Committee at the University of Southampton (ERGO II 56025.A3). A total of 31 adults (aged 18 to 70 years) participated in the study. In 22 subjects, pure-tone hearing thresholds (PTHTs) were below 20 dB HL for 250, 500, 1000, 2000, 4000, and 8000 Hz tones, indicating normal hearing. The remaining nine subjects had varying degrees of hearing loss: four with mild hearing loss (20 > PTHT ≤ 40 dB nHL), one with moderate hearing loss (40 > PTHT ≤ 70 dB nHL), one with severe hearing loss (70 > PTHT ≤ 90 dB nHL) and two with profound hearing loss (PTHT > 90 dB nHL). Standard otoscopy and tympanometry examinations were also carried out.

For the ABR test, the aim was to estimate ABR hearing thresholds for 500, 1000, 2000, and 4000 Hz narrow-band CE-Chirps (Elberling & Don 2010). Chirp stimuli were generated using in-house Matlab software, and were calibrated using a 94 dB SPL calibration piston, a Brüel and Kjaer type 2112 sound level meter, and an oscilloscope. The peak-to-peak amplitude of the 94 dB SPL calibration piston was initially measured using the oscilloscope to establish a reference point. Chirp calibration in dB hearing level (HL) was then performed using peak-to-peak amplitude values given in the International Organization for Standardization 389-6: 2007 in conjunction with the UK National Hearing Screening Protocol recommended stimulus reference levels for ABRs.

During the ABR test, subjects reclined in a comfortable chair in a quiet room and were asked to relax with their eyes closed. Chirps were then presented via an RME Fireface UC soundcard through ER-2 insert phones at a rate of 47.17 Hz, and data were recorded using an Interacoustics Eclipse system with electrodes placed at the vertex (active electrode), the nape of the neck (reference), and mid-forehead (ground). Line-level EEG signals were then routed back to Matlab via the RME Fireface at a sampling rate of 48 kHz, after which they were downsampled (after appropriate anti-alias filtering) to 5 kHz and band-pass filtered from 30 to 1500 Hz using a sixth-order Butterworth filter. Artifact rejection was also applied using a ±20 µV rejection level. Subjects were offered breaks in between test protocols, and additional breaks were permitted if requested.

In each subject, the ABR audiogram was estimated twice, once using GPs with active learning (see ABR audiogram estimation using GPs and active learning) and once using visual inspection by examiners (see Visual inspection by examiners). In addition, behavioral ABR hearing thresholds were estimated using a standard 10-down-5-up approach. It is worth emphasizing that behavioral testing was carried out last to avoid biasing the visual inspection results. The behavioral hearing thresholds were taken as the gold standard, and were used to assess the accuracy of the GP- and BSA-estimated hearing thresholds in the sections later.

Approximately 4 hr were allocated for the full test procedure. However, in cases where subjects gave noisy signals, there was not always sufficient time to estimate hearing thresholds for all four chirp stimuli, particularly when using visual inspection by examiners. Table 1 shows the total number of estimated hearing thresholds for each stimulus when using GPs with active learning and visual inspection by examiners who followed guidelines provided by the BSA.

**TABLE 1.** The number of estimated ABR hearing thresholds when using the GP approach and visual inspection by examiners who followed guidelines provided by the British Society of Audiology (2019)

	GP					BSA				
	0.5 kHz	1 kHz	2 kHz	4 kHz	Sum	0.5 kHz	1 kHz	2 kHz	4 kHz	Sum
HI	8	8	8	8	32	8	7	6	7	28
NH	22	22	22	22	88	11	14	18	21	64
Sum	30	30	30	30	120	19	21	24	28	92

The number of hearing threshold estimates are shown separately for NH and HI subjects, per stimulus. The BSA approach usually initiated testing with the 4 kHz chirp, hence the higher number of hearing threshold estimates for this stimulus. This contrasts with the GP approach, which estimates hearing thresholds for all four frequencies simultaneously, hence the equal number of hearing threshold estimates at each frequency when using GPs. The table also shows the total number of estimated thresholds, summed across frequencies as well as NH and HI listeners.

BSA, British Society of Audiology; GP, Gaussian process; HI, hearing-impaired; NH, normal-hearing.

#### Posterior Auricular Muscle Artifacts

It is important to note that in the initial piloting phase, the reference electrode was placed on the right mastoid, as opposed to the nape of the neck. At high stimulus levels, this led to posterior auricular muscle artifacts, which adversely affects the regression analysis conducted by the GP. This issue was overcome by moving the reference electrode to the nape of the neck.

### ABR Audiogram Estimation Using GPs and Active Learning

GPs with active learning were previously described in Chesnaye et al. (2023) for ABR hearing threshold estimation at a single frequency. In what follows, the approach is adapted for ABR audiogram estimation, that is, hearing threshold estimation at multiple frequencies. The overarching aim for the GP is to infer hearing threshold from the amplitude-intensity growth function, defined as the ABR wave V peak-to-trough amplitude (PTTa) across stimulus levels. The following sections describe this process in detail.

#### Peak-to-Trough Amplitude Estimation

The initial challenge is to estimate the PTTa values, which later serve as the data inputs for the GP. Due to the low signal to noise ratio (SNR) of the ABR, many waveforms (each time-locked to a stimulus), are averaged to reduce “noise,” giving what is known as the coherent average. The coherent average is then further inspected for PTTa estimation, which involves locating the wave V peak and trough, and then computing the difference.

One challenge with PTTa estimation is variability in ABR peak and trough latencies due to factors such as the stimulus level, stimulus frequency, subjects’ hearing ability, and individual physiology (Picton 2011). To ensure that the peak and trough can be located, a relatively wide search window is needed. However, using a wide search window increases the probability of detecting spurious peaks and troughs, which introduces noise to the PTTa estimates. There is hence a trade-off between maintaining a narrow search range to minimize noise, and broadening the search range to ensure reliable peak and trough detection. As a compromise, a sliding window approach was adopted, which constrains the search interval by assuming the peak precedes the trough, and that the time interval between peak and trough is less than 8 msec. Further details on the approach can be found in Chesnaye et al. (2023) and are also presented in Supplementary Digital Content 1, http://links.lww.com/EANDH/B477, in the present work.

It is also important to note that the residual background activity in the coherent average introduces a bias to the PTTa estimates, which adversely affects the regression analysis conducted by the GP. This bias is impacted by the ABR’s SNR, making it difficult to estimate, which previously incentivized a maximum likelihood approach for unbiased PTTa estimation. The maximum likelihood approach aims to replace the biased PTTa estimates with unbiased estimates. The approach uses bootstrapping to approximate the expected distribution of the biased PTTa values under a range of unbiased PTTa values, after which the likelihood can be generated that some observed (biased) PTTa value arose under each distribution. This then gives a distribution over unbiased PTTa values, which is inspected to determine the most likely unbiased PTTa value. This distribution is also used to generate a variance for the unbiased PTTa estimate. A more comprehensive description can again be found in Chesnaye et al. (2023) as well as Supplementary Digital Content 1, http://links.lww.com/EANDH/B477, in the present work.

In what follows, the (unbiased) PTTa estimates will be denoted by $o_{X_{L},X_{F}}$, and the corresponding variances by ${\sigma^{2}}_{X_{L},X_{F}}$ where $x_{L}$ and $x_{F}$ indicate the stimulus level (in dB HL) and stimulus frequency (in Hz), respectively. The $o_{X_{L},X_{F}}$ and ${\sigma^{2}}_{X_{L},X_{F}}$ values serve as the inputs for the GP, as described later.

#### Gaussian Processes

The function to estimate by GP is the (unbiased) PTTa value as a function of the stimulus level and frequency, henceforth denoted by $f\left(x_{L},x_{F}\right)$. The following section provides a detailed overview of this estimation process.

##### Defining the prior

When delving into the estimation process, it is helpful to consider the GP as a model of our beliefs regarding $f\left(x_{L},x_{F}\right)$. Before having collected data, there is typically much uncertainty surrounding the $f\left(x_{L},x_{F}\right)$ values, but there is also prior knowledge available, that is, it is known that PTTa values usually range from 0 to ~1 µV, and that PTTa values are similar across adjacent levels and frequencies (Picton et al. 1981; Nousak & Stapells 2005; Picton 2011). This prior knowledge is used to define an initial set (i.e., a distribution) of expected growth functions, known as the GP prior.

More specifically, the GP prior is defined by a multivariate normal (MVN) distribution, which is, in turn, defined by a mean vector and a covariance matrix (defined later). The mean vector represents the most likely PTTa value at each stimulus level ($x_{L}$) and frequency ($x_{F}$). Uncertainty regarding the most likely PTTa values is then encoded through the main diagonal of the covariance matrix, representing the variance of the MVN distribution. In addition, the off-diagonal elements of the covariance matrix are used to encode expectations of “function smoothness,” that is, the extent to which PTTa values are similar across stimulus levels and frequencies, further clarified later.

**Fig. 1.**
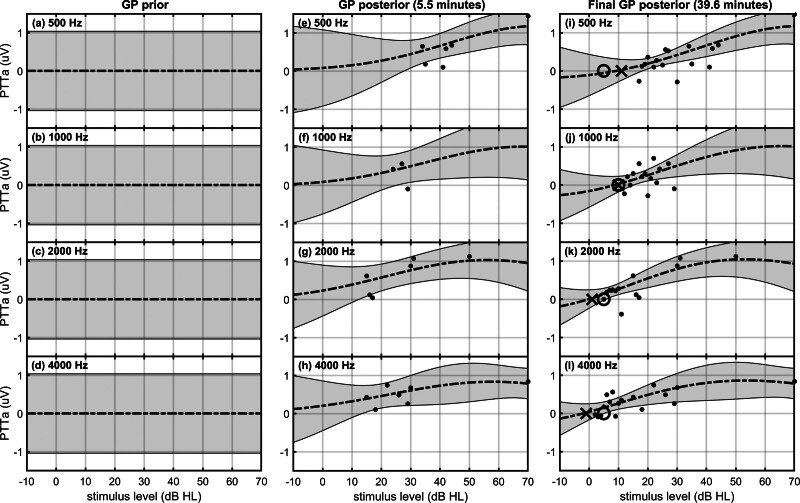
An illustration of the GP approach for ABR hearing threshold estimation for 500, 1000, 2000, and 4000 Hz chirp stimuli in a single test subject. Panels (A–D) show simplified depictions of the GP prior, which includes the mean (thick dashed line) and 99% CIs (shaded regions) of an MVN distribution. Panels (E–H) show the GP posterior after having estimated PTTa values at several stimulus levels and frequencies, indicated by small dots. Panels (I–L) show the GP posterior after data collection was stopped and hearing threshold inferred. For this subject, the estimated hearing thresholds were 11, 10, 1, and −1 dB HL for 500, 1000, 2000, and 4000 Hz chirps (indicated by **X**), respectively, which coincided closely with the behavioral hearing thresholds, equal to 5, 10, 5, and 5 dB HL (indicated by **O**). Further details are provided in the main text. ABR indicates auditory brainstem response; CI, confidence interval; GP, Gaussian process; MVN, multivariate normal; PTTa, peak-to-trough amplitude.

To illustrate with an example, consider panels (A–D) in Figure 1: the thick dashed line is the mean vector, representing the most likely $f\left(x_{L},x_{F}\right)$ values at 500 Hz (panel A), 1000 Hz (panel B), 2000 Hz (panel C), and 4000 Hz (panel D). The shaded regions then represent ±2.575 SDs (the 99% confidence intervals) from the mean, representing the level of uncertainty surrounding the mean vector. Note that panels (A–D) are simplified depictions of a single, high-dimensional MVN distribution, and show just the MVN mean and 99% confidence intervals. Function smoothness across level and frequency is not immediately evident from panels (A–D), but becomes apparent after having observed data.

When defining the GP prior, it is first necessary to specify the stimulus levels and frequencies along which the prior is defined. These locations are referred to as the “prediction locations,” as this is where the GP aims to predict the $f\left(x_{L},x_{F}\right)$ function values. The prediction locations are denoted by **X**_**P**_ with elements $[x_{1}$, $x_{2}$, …, $x_{p}]$ where each $x_{k}$ element (for *k* = 1, 2, …, *p*) is a vector that specifies the level and frequency, denoted by $x_{L}$ and $x_{F}$ respectively, for the stimulus at the *k*th prediction location. In the present work, $x_{L}$ took integer values ranging from −10 to 70 dB HL (81 in total) and $x_{F}$, was set to either 500, 1000, 2000, or 4000 Hz, giving a total of 81 × 4 = 324 prediction locations.

After specifying the prediction locations, the mean vector and covariance matrix for the GP prior are defined. The mean vector, say $\mu_{p}$, was set to zero for all prediction locations, giving $\mu_{p}\left(x_{L},x_{F}\right)$ = 0 for all $\left[x_{L},x_{F}\right]\in$ **X**_**P.**_ A zero-mean prior essentially represents the initial belief that the subject is deaf, which might be viewed as a clinically conservative starting position, that is, it may be safer to assume that a careful assessment of hearing function is required, rather than assuming the subject has normal hearing. As discussed in Chesnaye et al. (2023), a zero-mean prior also facilitates monotonic estimates of the growth function, which then provides directional guidance on where hearing threshold is located, resulting in a more efficient test.

With respect to the covariance matrix, say $\Sigma_{P}$, this can be specified through a covariance function (Rasmussen & Williams 2006). It was assumed that PTTa values at adjacent stimulus levels and frequencies were similar, and that similarity decreases with the distance in level and frequency, which can be modeled using an exponential covariance function (Gramacy 2021):


$$\begin{array}{l} \Sigma_{P}=cov\left(f\left(x_{L1},x_{F1}\right),f\left(x_{L2},x_{F2}\right)\right)=s\cdot e^{-\frac{|x_{L1}-x_{L2}|}{\theta_{dB}}-\frac{|\log x_{F1}-\log x_{F2}|}{\theta_{Hz}}} \end{array}$$ (1)

which is defined for all $\left[x_{L1},x_{F1}\right]\in X_{P}$ and all $\left[x_{L2},x_{F2}\right]\in X_{P}$. With respect to the scale parameter *s*, this specifies the main diagonal of the covariance matrix, which encodes the level of uncertainty surrounding the mean vector, and thus the width of the prior in Figure 1. Following Chesnaye et al. (2023), it was assumed that 99.9% of PTTa values were smaller than 1.25 μV, which was motivated by findings from the literature (Picton et al. 1981; Nousak & Stapells 2005). The two-sided 99.9% confidence intervals are given by ±3.09 SDs from the mean, giving $s={\left(\frac{1.25}{3.09}\right)}^{2}=0.1636\mu V$.

With respect to the $\theta_{dB}$ and $\theta_{Hz}$ length scale parameters, these are used to encode expectations of function smoothness with larger values indicating smoother functions. One complication when specifying these parameters is that growth functions differ across subjects depending on, for example, hearing ability, which implies that the optimal $\theta_{dB}$ and $\theta_{Hz}$ parameters may be subject-dependent. Therefore, rather than assume $\theta_{dB}$ and $\theta_{Hz}$ in advance, these were estimated from the data using standard maximum likelihood estimation (Gramacy 2021). The $\theta_{dB}$ length scale was confined to the (1000, 2000) interval as this previously showed favorable results (Chesnaye et al. 2023). Results from a pilot study (specifics not presented) also suggested a favorable test performance when confining $\theta_{Hz}$ to the (0.05, 1) interval.

##### Deriving the posterior

As data becomes available, our beliefs (and our level of confidence) regarding the $f\left(x_{L},x_{F}\right)$ function values change, which is accounted for by transforming the GP prior into a GP posterior. This transformation depends on the observed data (the $o_{X_{L},X_{F}}$ values) and on how noisy the data are (the ${\sigma^{2}}_{X_{L},X_{F}}$ values), but also on prior assumptions, particularly assumptions regarding “function smoothness.” The latter leads to information being “smeared” across adjacent levels and frequencies, which helps to reduce uncertainty regarding the expected $f\left(x_{L},x_{F}\right)$ function values.

To again illustrate with an example, consider panels (E–H) in Figure 1, which show the MVN mean and 99% confidence intervals of a GP posterior after having observed PTTa values at several test locations (indicated by dots in the figure). Note that the $o_{L,F}$ estimates are noisy (the ${\sigma^{2}}_{X_{L},X_{F}}$ values are nonzero), which implies that there is still uncertainty regarding the true $f\left(x_{L},x_{F}\right)$ values at the test locations. It is now also evident how function smoothness impacts on expectations of $f\left(x_{L},x_{F}\right)$: uncertainty was reduced not just at the test locations, but also at the adjacent levels and frequencies.

More formally, data are collected by probing $f\left(x_{L},x_{F}\right)$ at a set of test locations, denoted by **X**_**T**_ with elements $[x_{1}$, $x_{2}$, …, $x_{T}]$ where $x_{k}$ (for *k* = 1, 2, …, T) is again a vector, now containing the [$x_{L},x_{F}$] values associated with *k*th test location. It is worth emphasizing that the **X**_**T**_ test locations may differ from the **X**_**P**_ prediction locations. Probing $f\left(x_{L},x_{F}\right)$ at **X**_**T**_ thus gives a T-dimensional vector of PTTa values, say **O**_**T**_, with associated variances $\vartheta_{T}^{2}$. The posterior mean vector can then be generated using (Gramacy 2021):

$$\begin{array}{l} {\bar{\mu }}_{P}=\mu_{P}+\Sigma_{PT}{\left[\Sigma_{T}+I_{T}\vartheta_{T}^{2}\right]}^{-1}\left(O_{T}-\mu_{T}\right) \end{array}$$ (2)

where $I_{T}$ is a T-dimensional identity matrix, $\mu_{T}\left(x_{L},x_{F}\right)$ denotes the prior mean for the $X_{T}$ test locations and was set to 0 for all $\left[x_{L},x_{F}\right]\in X_{T}$, $\Sigma_{T}$ is the prior covariance matrix for $X_{T}$, and $\Sigma_{PT}$ is the prior cross-covariance matrix between $X_{P}$ and $X_{T}$. Note that $\Sigma_{T}$ is specified using Eq. (1) for all $\left[x_{L1},x_{F1}\right]\in X_{T}$ and all $\left[x_{L2},x_{F2}\right]\in X_{T}$, giving a T × T dimensional covariance matrix. Similarly, $\Sigma_{PT}$ is specified using Eq. (1), now for all $\left[x_{L1},x_{F1}\right]\in X_{P}$ and all $\left[x_{L2},x_{F2}\right]\in X_{T}$, giving a P × T dimensional covariance matrix.

Last, the GP posterior covariance matrix is given by (Gramacy 2021):

$$\begin{array}{l} {\bar{\Sigma }}_{P}=\Sigma_{P}-\Sigma_{TP}{\left[\Sigma_{T}+I_{T}\vartheta_{T}^{2}\right]}^{-1}\Sigma_{PT} \end{array}$$ (3)

where $\Sigma_{TP}$ is the prior cross-covariance matrix between $X_{T}$ and $X_{P}$, which is again specified using Eq. (1), now for all $\left[x_{L1},x_{F1}\right]\in X_{P}$ and all $\left[x_{L2},x_{F2}\right]\in X_{T}$. The GP posterior was re-computed every 500 epochs (approximately every 10 sec). This involves updating $O_{T}$ and $\vartheta_{T}^{2}$ to include the new measurement, and if the test location is new (i.e., it is not already contained by $X_{T}$), then $X_{T}$ is also updated along with the $\Sigma_{T}$, $\Sigma_{TP}$, $\Sigma_{PT}$, and $I_{T}$ matrices.

#### Active Learning Rules

The purpose of the active learning rules is to automatically adjust the stimulus, and efficiently locate hearing threshold at each frequency. The rules were previously described in Chesnaye et al. (2023) for hearing threshold estimation at a single frequency, and are briefly summarized later, with some adjustments to enable testing at multiple frequencies. In what follows, ABR hearing threshold for stimulus frequency $x_{F}$ refers to the lowest $x_{L}$ value where $f\left(x_{L},x_{F}\right)>0$, or equivalently the largest $x_{L}$ value where $f\left(x_{L},x_{F}\right)=0$ = 500, 1000, 2000, and 4000 Hz.

One challenge with hearing threshold estimation is that $f\left(x_{L},x_{F}\right)$ is zero not just at a single level, but for all inaudible stimuli, which introduces the risk of the GP converging on levels below hearing threshold. The active learning rules strive to mitigate this risk by approaching hearing threshold from the higher stimulus levels, that is, from the $f\left(x_{L},x_{F}\right)>0$ region (Chesnaye et al. 2023). To facilitate this, the GP first aims to locate several nonzero PTTa targets, including the *T*_1_ = 0.5, *T*_2_ = 0.3, *T*_3_ = 0.25, *T*_2_ = 0.2, and *T*_1_ = 0.15 μV targets. The GP starts with locating the largest target (i.e., *T*_1_) at each stimulus frequency, and only moves on to the smaller targets after having located the larger ones.

To locate a target, the GP first finds the most likely stimulus level where $f\left(x_{L},x_{F}\right)=T_{i}$ μV, per frequency. The most likely stimulus levels associated with target $T_{i}$ are denoted by $x_{T_{i}}$, which are found for $x_{F}$ = 500, 1000, 2000, and 4000 Hz using:

$$\begin{array}{l} x_{T_{i}}=arg\underset{x_{j}\in X}{\max}\;N\left(T_{i},{\bar{\mu }}_{\left[x_{j},x_{F}\right]},\sigma_{\left[x_{j},x_{F}\right]}^{2}\right) \end{array}$$ (4)

where **X** is a vector containing all potential stimulus levels to test for the given $x_{F}$, and $N\left(T_{i},{\bar{\mu }}_{\left[x_{j},x_{F}\right]},\sigma_{\left[x_{j},x_{F}\right]}^{2}\right)$ is a univariate GP posterior with mean ${\bar{\mu }}_{\left[x_{j},x_{F}\right]}$ and variance $\sigma_{\left[x_{j},x_{F}\right]}^{2}$, evaluated at location $T_{i}$. The ${\bar{\mu }}_{\left[x_{j},x_{F}\right]}$ and $\sigma_{\left[x_{j},x_{F}\right]}^{2}$ values are computed using Eqs. (2) and (3), respectively, but using a single prediction location, equal to $\left[x_{j},x_{F}\right]$.

Next, the SD of the GP posterior is inspected at each of the four [$x_{T_{i},}$, $x_{F}$] locations. Large SD values indicate uncertainty regarding the $T_{i}$ target locations, suggesting that additional data collection may be necessary, whereas small SD values indicate less uncertainty, which suggests that the $T_{i}$ target might have already been located. More specifically, a $T_{i}$ target was deemed located at frequency $x_{F}$ when the SD of the GP posterior at location [$x_{T_{i},}$, $x_{F}$] was less than some threshold value, given by *δ*_1_ = 0.2, *δ*_2_ = 0.15, *δ*_3_ = 0.1, *δ*_4_ = 0.075, and *δ*_5_ = 0.05 μV for *T*_1_, *T*_2_, *T*_3_, *T*_4_, and *T*_5_, respectively (Chesnaye et al. 2023). The next stimulus to test at was then specified using the [$x_{T_{i},}$, $x_{F}$] values where uncertainty along the GP posterior was largest.

Last, in some subjects with hearing loss, it is conceivable that the $f\left(x_{L},x_{F}\right)$ growth curve is smaller than the $T_{i}$ target for all test locations, in which case the GP may waste time trying to locate a target that does not exist. To mitigate this risk, the GP first inspects the GP posterior at some maximum test level, per frequency. The corresponding stimuli are specified using $\left[x_{Lmax},x_{F}\right]$, for $x_{F}$ = 500, 1000, 2000, and 4000 Hz. If the most likely PTTa value under the GP posterior for the $\left[x_{Lmax},x_{F}\right]$ stimulus is smaller than the $T_{i}$ target value, then the next stimulus to test at is instead specified using $\left[x_{Lmax},x_{F}\right]$. The most likely PTTa value at $\left[x_{Lmax},x_{F}\right]$ is given by:

$$\begin{array}{l} T_{Lmax,F}=arg\underset{a\in A}{\max}\;N\left(a,{\bar{\mu }}_{\left[x_{Lmax},x_{F}\right]},\sigma_{\left[x_{Lmax},x_{F}\right]}^{2}\right) \end{array}$$ (5)

where A is a vector, containing a range of potential PTTa values to evaluate, and $N\left(a,{\bar{\mu }}_{\left[x_{Lmax},x_{F}\right]},\sigma_{\left[x_{Lmax},x_{F}\right]}^{2}\right)$ is a univariate GP posterior with mean ${\bar{\mu }}_{\left[x_{Lmax},x_{F}\right]}$ and variance $\sigma_{\left[x_{Lmax},x_{F}\right]}^{2}$, evaluated at location a. The ${\bar{\mu }}_{\left[x_{Lmax},x_{F}\right]}$ and $\sigma_{\left[x_{Lmax},x_{F}\right]}^{2}$ values can again be computed using Eqs. (2) and (3), respectively, but using a single prediction location, now equal to $\left[x_{Lmax},x_{F}\right]$. After having located all $T_{i}$ targets, data collection was stopped, and hearing thresholds were inferred, achieved using Eq. (4) with $T_{i}=0$ for $x_{F}$ = 500, 1000, 2000, and 4000 Hz.

### Visual Inspection by Examiners

To establish a rough benchmark to compare against, subject ABR audiograms were also estimated in real time through visual inspection by 2 examiners. These examiners were Audiology undergraduates with prior experience in inspecting ABR waveforms. In order to mitigate examiner bias and ensure a sufficiently accurate test outcome, the examiners were asked to follow guidelines provided by the BSA (British Society of Audiology 2019). These guidelines (further described later) provide a rigorous set of rules for determining the presence or absence or ABRs, but were previously formulated for ABR detection in sleeping infants where data tend to be less noisy than non-sleeping adults. Consequently, the criteria for determining ABR absence were deemed too strict in the present work, and a minor adjustment was introduced, as described later.

#### Visual Inspection Interface

Data were presented to the examiners as two replicates of the coherently averaged epoch using an in-house Matlab interface. The first replicate was given by the average of the odd-numbered epochs (epochs 1, 3, 5, etc) and the second by the average of the even-numbered epochs (epochs 2, 4, 6, etc). The 0 to 20 msec poststimulus interval of the coherent average replicates was plotted in μV with a user-adjustable *y* axis. To aid hearing threshold estimation, a second panel was also included, which displayed the full threshold series for the stimulus frequency in question, showing all measured coherent average replicates in descending dB HL order. Illustrations of the Matlab interface are provided in Supplementary Digital Content 2, http://links.lww.com/EANDH/B478.

#### Clear Response Criteria

The BSA criteria for concluding that a “clear response” (CR) was present is that coherent average replicates show a high degree of similarity while also exhibiting the expected waveform characteristics in terms of amplitude, latency, and morphology (British Society of Audiology 2019). A further criterion is that the wave V PTTa should exceed 40 nV and should be at least three times larger than the residual background activity. The residual background activity was estimated by visually evaluating the difference between the two coherent average replicates.

#### Response Absent Criteria

The BSA criteria for concluding “response absent” (RA) is first that the criteria for CR were not met. The criteria also state that the coherent average replicates should be “appropriately flat” with “no evidence of a response,” and the residual background activity should be less than, or equal to, 25 nV. The 25 nV noise requirement, however, was deemed too strict for adult ABR data, requiring impractically large ensemble sizes (i.e., tens of thousands of epochs) before being met. This criterion was therefore replaced with the requirement that at least 10,000 epochs were averaged (5000 per coherent average replicate) before RA was concluded. A maximum ensemble size of 20,000 epochs was also specified: if CR or RA was still not concluded after recording 20,000 artifact-free epochs, then RA was concluded by default.

#### Stimulus Selection Protocol

The examination was initiated with a 4 kHz 50 dB HL chirp, and data were analyzed (and results updated) every ~10 sec. For each stimulus frequency, examiners aimed to locate hearing threshold using a 10-down-10-up approach, that is, the stimulus level was decreased by 10 dB if CR was concluded, or increased by 10 dB if RA was concluded. Hearing threshold was inferred after determining both RA and CR, and was assumed to be the lowest level where CR was identified.

## RESULTS

**Fig. 2.**
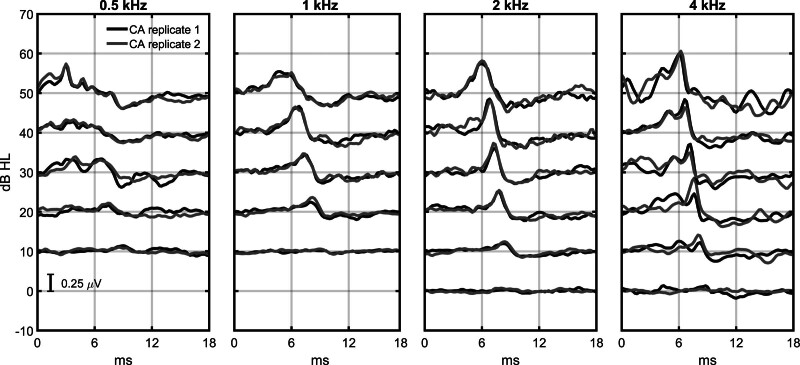
An example of an ABR threshold series that was inspected visually by an examiner. ABR hearing thresholds (estimated by the examiner) in this subject were 20, 20, 10, and 10 dB HL for the 0.5, 1, 2, and 4 kHz chirps, respectively, and the corresponding behavioral hearing thresholds were 5, 10, 5, and 5 dB HL, respectively. Test times for this subject were 13.1, 10.5, 10.5, and 24.6 min (respectively), giving an overall test time of 58.7 min. Note that the examiners inspected the ABR waveforms using a Matlab interface, which displayed the waveforms differently than shown. Illustrations of the Matlab interface are provided in Supplementary Digital Content 2, http://links.lww.com/EANDH/B478. ABR indicates auditory brainstem response.

**Fig. 3.**
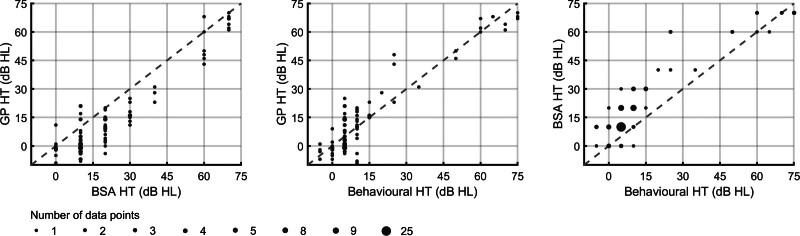
The estimated hearing thresholds are presented as scatter plots. The left panel shows the GP HT against the BSA HT. The middle panel shows GP HT against the behavioral HT, and the right panel shows the BSA HT against the behavioral HT. BSA indicates British Society of Audiology; GP, Gaussian process; HT, hearing threshold.

**Fig. 4.**
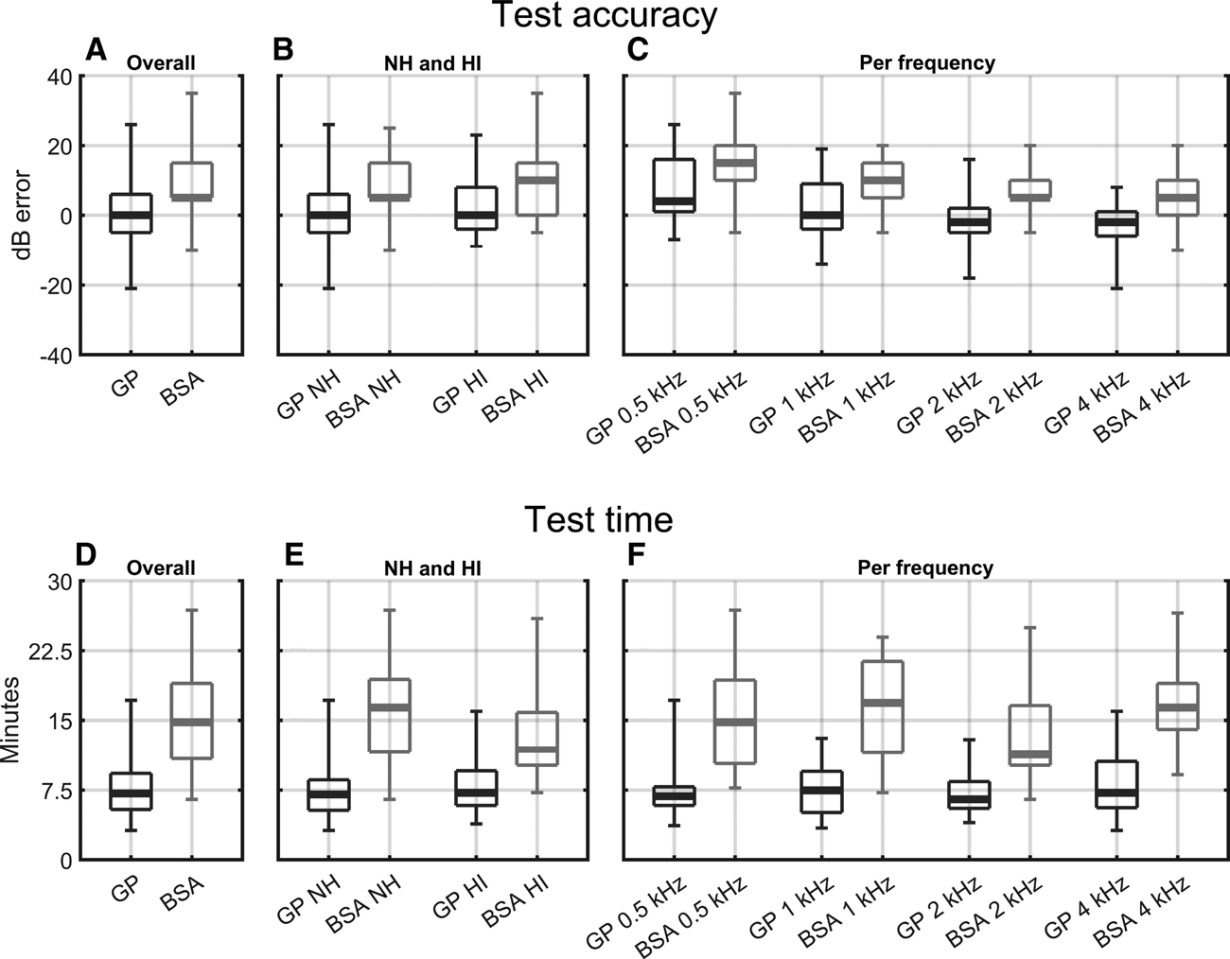
Test accuracies and test times, per frequency, from the subject data analysis, presented as box and whisker plots. Panels (A–C) show the dB estimation errors, defined as the BSA- or GP-estimated hearing thresholds, whereas panels (D–F) show the test times, that is, the number of preprocessed and artifact-free epochs (presented in minutes) required for hearing threshold estimation. Panels (A–D) furthermore show an “Overall” comparison, which implies that no distinction was made between NH and HI subjects, nor between stimulus frequencies tested. This contrasts with panels (B and E) where a distinction was made between NH and HI subjects, but not between frequencies, and with panels (C and F) where a distinction was made between frequencies, but not between NH and HI subjects. BSA indicates British Society of Audiology; GP, Gaussian process; HI, hearing-impaired; NH, normal-hearing.

Test performance was evaluated in terms of test accuracy and test time. Test accuracy was assessed using the “dB estimation error,” defined as the GP- or BSA-estimated hearing threshold minus the behavioral hearing threshold, and test time was assessed using the number of preprocessed (and artifact-free) epochs required for hearing threshold estimation, per frequency. An example of an ABR threshold series that was inspected by the examiners is shown in Figure 2. The GP, BSA, and behavioral hearing thresholds are also presented in scatter plots in Figure 3, and box and whisker plots of the errors and test times are presented in Figure 4. These results are further evaluated in the sections later.

### Grand Comparison

First, an overall, or “grand comparison” between the GP and BSA methods was drawn. For this comparison, no distinction was made between NHNH and HI individuals, nor between stimulus frequencies. In the GP, 120 ABR hearing thresholds were estimated in 30 subjects, whereas in the BSA approach, 92 thresholds were estimated in 31 subjects (see also Table 1). The resulting dB estimation errors and test times are shown as box and whisker plots in Figure 4, panels (A and D), respectively. The median estimation error was 0 dB for the GP and 5 dB for the BSA approach, whereas the median test time was 7.1 min for the GP and 14.8 min for BSA. The GP thus showed less bias in the estimated hearing thresholds and a reduced median test time of ~50%. The GP also demonstrated a smaller spread in test times, that is, the SD of the test times (per threshold estimate) was 2.84 min for the GP versus 5.03 min for the examiners. However, the spread of the dB estimation errors, which is arguably more important than the median estimation error, was slightly larger for the GP, that is, the STD of the estimation errors was 8.87 dB, whereas for the examiners this was 8.03 dB.

Post hoc statistical analysis was carried out to test if the differences in test times and test accuracies between the GP and BSA methods were statistically significant. A complication, however, is that the GP’s results are correlated across frequencies due to the “smearing effect” described in Materials and methods. This implies that the number of independent data points is unknown. To facilitate the post hoc comparison, results were therefore first combined across frequencies to obtain independent data points, as described later.

#### Comparing Test Times

For each method and for each test subject, a mean test time was computed by averaging the individual test times for the frequencies tested (four maximum). For the GP, this resulted in 30 mean test times with a grand mean test time of 7.6 min, whereas for the BSA approach, this led to 31 mean test times with a grand mean test time of 15.5 min. Data were approximately normally distributed and were compared using a two-sample two-tailed *t* test, giving *p* < 0.001, with an effect size (measured using Cohen d) of *d* = 2.467.

#### Comparing Test Accuracies

A similar approach was used to compare the dB estimation errors, except that the absolute values of the estimation errors were taken before averaging across frequencies. For the GP, 30 “mean absolute estimation errors” were computed with a grand mean absolute error of 6.5 dB, whereas for the BSA approach, 31 “mean absolute estimation errors” were computed with a grand mean absolute error of 9.8 dB. The grand mean absolute errors were deemed significantly different (two-sample two-tailed *t* test; *p* = 0.021, *d* = 0.6248).

#### Comparison of Thresholds

The relationships between the GP, BSA, and behavioral hearing thresholds are displayed using scatter plots in Figure 3. Visually inspecting the results suggests that the GP-estimated thresholds were unbiased relative to the behavioral hearing thresholds, and that the BSA-estimated hearing thresholds were slightly overestimated relative to the behavioral as well as the GP-estimated thresholds.

### Effect of Hearing Ability

This comparison aims to test whether hearing loss impacted on the GP’s and/or the BSA’s test performance. The dB estimation errors and test times are now presented separately for NH and HI subjects in Figure 4, panels (B and E). For the GP approach, the median dB errors were 0 dB (NH) and 0.5 dB (HI), whereas in the BSA approach these were 8 dB (NH) and 9.3 dB (HI). The GP’s median test times were 7.1 (NH) and 7.3 (HI) min, whereas BSA’s median test times were 16.5 (NH) and 12.2 (HI) min. Data were again combined across frequencies to obtain independent data points, as described in Grand comparison. Results showed no significant differences between the NH and HI test conditions (two-sample two-tailed *t* tests; *p* > 0.05).

### Effect of Frequency

For the third and final analysis, the aim was to test if stimulus frequency impacted on the GP’s or BSA’s test performance. The dB estimation errors and test times are now presented separately for each stimulus frequency in panels (C and F) in Figure 4. No distinction was made between NH and HI individuals. The median dB errors for the GP were 4, 0, −2, and −2 dB for the 500, 1000, 2000, and 4000 Hz chirps, and the corresponding median test times were 7, 7.5, 6.5, and 7.4 min. The median dB errors for the BSA approach were 15, 10, 5, and 5 dB for the 500, 1000, 2000, and 4000 Hz chirps, and the corresponding median test times were 14.8, 16.9, 11.4, and 16.6 min. For the GP approach, hearing thresholds were estimated for all four frequencies in 30 subjects, whereas for the BSA, all four thresholds were estimated in just 11 subjects due to time constraints. Test times and estimation errors were now treated as repeated measurements across frequencies. Results from Friedman test show that frequency significantly impacted on the dB errors for the GP (*p* < 0.001) and the BSA approach (*p* = 0.025) with a trend that error reduces as frequency increases. Frequency also impacted on the GP’s test times (*p* = 0.025) but not on the BSA’s test times (*p* = 0.241), which might be due to the smaller sample size.

## DISCUSSION

Research aimed at improving the efficiency and accuracy of ABR hearing tests using objective detection methods has traditionally focused on “null hypothesis significance testing,” that is, evaluating the hypothesis of “no ABR present.” Many of these methods were also designed with ABR hearing threshold and/or audiogram estimation in mind. A limitation, however, is that the majority of these methods were evaluated under simplified test conditions, and generally disregard the sequential testing aspects involved in clinical applications.

When used in the clinic, ABR detection methods are typically applied repeatedly to the accruing data over time, known as a sequential test. Sequential tests are important for providing timely feedback to clinicians, but also for keeping test time low as data collection can be stopped early in the case of a CR. The challenge is that repeated hypothesis testing inflates the FPR (Armitage et al. 1969), and to control the significance level of the test, the critical thresholds for response detection need to be chosen carefully (Stürzebecher et al. 2005; Stürzebecher & Cebulla 2013; Cebulla & Stürzebecher 2015; Chesnaye et al. 2019, 2020; Zanotelli et al. 2020).

For ABR hearing threshold estimation, sequential testing comes into play when determining the presence or absence of an ABR at each stimulus level, but also when adjusting the stimulus level to locate the hearing threshold. The most common clinical approach uses an X-down-Y-up test strategy, which implies that the stimulus level is decreased by X dB following a detection, or increased by Y dB following a non-detection (Özdamar et a. 1990). Note that this further increases the number of hypothesis tests carried out, thus exacerbating the issue of inflated FPRs.

Considering the vast number of objective detection methods in the literature, it is surprising that just a handful of authors have evaluated test performance under “fully sequential test conditions,” that is, sequential data analysis when determining ABR present/absent for a single stimulus and when switching between levels to home in on hearing threshold. This was already recognized back in 1990 when Özdamar et al. (1990) stated, “*Contrary to the abundance of response recognition methods, little research has been done to develop such tracking algorithms*,” where “tracking algorithms” refer to stimulus selection protocols for locating hearing threshold. Building on work from Salvi et al. (1987), Özdamar et al. proceed to evaluate three X-down-Y-up test strategies, including a conventional 10-down-5-up approach, a 10-down-10-up Békésy test strategy, and the Parameter Estimation by Sequential Testing approach, which halves the step size with each change in stimulus level direction, starting with 20 dB steps and ending with 5. The lowest test times and smallest estimation errors were observed for the Parameter Estimation by Sequential Testing approach, which approximated behavioral hearing thresholds to within ~6.5 dB.

Although the study in Özdamar et al. (1990) considered sequential testing across stimulus levels, the detection methods—which included variance ratios and correlation coefficients—were still applied as single shot tests, that is, sequential testing for determining ABR present/absent at each stimulus level was not considered. Indeed, to the best of the authors' knowledge, objective methods for ABR hearing threshold estimation have been evaluated under fully sequential test conditions in just two publications (Berninger et al. 2014; Wang et al. 2021).

Starting with Berninger et al. (2014), ABR detection was carried out using the correlation coefficient (CC), computed between two coherent average replicates. The test starts by collecting data at a relatively high stimulus level, and if the CC exceeds 0.7, then an ABR is deemed present and the stimulus level is decreased. The procedure repeats until an ABR is deemed absent, which is concluded after reaching some maximum sample size without having detected an ABR (CC < 0.7). Two variations of this approach were also explored (for details, see Berninger et al. 2014). Results show average hearing threshold estimates ranging from 3.9 to 6.5 dB nHL, demonstrating test accuracies similar to those from Özdamar et al. (1990).

In Wang et al. (2021), response detection was carried out using cross-correlation functions, computed between three coherent average replicates. The test again starts at a relatively high stimulus level, and an ABR was deemed present if the time lags associated with the maximum peaks in the cross-correlation functions were smaller than ±0.3 msec. If an ABR is detected, then the stimulus level is decreased, and the procedure repeats until two consecutive non-detections are observed, which is concluded if the maximum sample size is reached without having detected an ABR. The approach was evaluated in mouse and human ABR threshold series, where it estimated hearing thresholds to within ±10 dB of visually identified thresholds (with a mean difference of 4.6 dB).

Various additional studies have investigated ABR hearing threshold estimation procedures, but these were applied either as post hoc tests, that is, after all, data have been collected (Vannier et al. 2002; Schilling et al. 2019; Suthakar & Liberman 2019; Thalmeier et al. 2022), or like Özdamar et al. (1990), utilizing single shot test strategies (Bogaerts et al. 2009). Starting with the latter, Bogaerts et al. (2009) use the ABR’s peak amplitude as test statistic, and an ABR is deemed present if the peak amplitude exceeds four times the SD of the background activity. The test starts at a high level, and if an ABR is detected, the level is decreased, until an ABR is deemed absent. The approach was evaluated in click- and tone-evoked ABR data in mice, and the accuracy of the estimated hearing thresholds was comparable to those obtained through visual inspection by clinicians.

In Vannier et al. (2002), a correlation-based threshold-seeking procedure built around an optimizable ABR template was proposed. This optimizable template was fine-tuned to maximize correlation with the coherently averaged epoch and was additionally constrained to preserve monotonicity in ABR waves I, II, III, and IV/V latencies across stimulus levels. This interesting approach requires multiple critical thresholds for response detection to be defined, along with a complex set of rules for fine-tuning test performance, which might raise concerns regarding overfitting and generalizability. Results show an average hearing threshold estimation error of 5 dB (SD 8.3 dB).

Moving on to methods in Schilling et al. (2019), Suthakar and Liberman (2019), and Thalmeier et al. (2022), these approaches are similar to the GP in the present work in that they aim to infer hearing threshold from the estimated ABR amplitude-intensity growth function, or some representation of it. In Suthakar and Liberman, the amplitude-intensity growth function was represented by a cross-correlation coefficient growth curve, and was estimated using power functions and sigmoid functions, whereas in Schilling et al., the growth function was represented by the root-mean-square growth curve, which was estimated using hard sigmoid functions. Last, in Thelameier et al., a self-supervised random forest regression model was used to predict sound intensity levels in a threshold series, followed by the fitting of a piece-wise function consisting of a constant element and a fourth-order polynomial. As mentioned previously, these methods were evaluated as post hoc tests, but might be adapted and/or evaluated for online sequential data analysis in future work.

### GPs for Hearing Threshold Estimation: Pros, Cons, and Study Limitations

The GP approach in the present work is attractive for online ABR hearing threshold estimation in the clinic, first because it no longer utilizes repeated null hypothesis significance testing, and instead focuses on parameter estimation. This implies that complex sequential tests (Stürzebecher et al. 2005; Chesnaye et al. 2020; Zanotelli et al. 2020) for controlling FPRs are no longer required. It is worth noting that these sequential tests require the number of hypothesis tests, as well as the sample size for each test, to be specified at the outset. If the maximum test time is reached without having inferred ABR present/absent (and/or having located hearing threshold), then the test cannot be prolonged without inflating the FPR. This contrasts with the GP where data collection continues until the desired level of confidence has been obtained, which is specified in the present work through the δ_i_ thresholds. Note that although these thresholds are fixed at the outset, the GP’s test time is still adaptive: when data are noisy, data collection is automatically prolonged, and vice versa for less noisy data. This facilitates quality control and helps to bring the examination to an unambiguous outcome in terms of hearing threshold location. A challenge remains, however, in how to choose these δ_i_ values, as well as the Ti targets for the GP to estimate. These parameters impact on the GP’s stimulus selection decisions but have not yet been fully optimized. As mentioned in Chesnaye et al. (2023), numerous rule sets for stimulus selection can be envisioned, and it is highly likely that the rules adopted in the present work are suboptimal.

One of the main advantages of the GP over conventional detection methods is that it can learn and exploit the correlation structure underlying the ABR waveforms through the $\theta_{dB}$ and $\theta_{Hz}$ length scale parameters. These correlations hold valuable information, but are neglected by most detection methods in the literature, albeit with some exceptions (Vannier et al. 2002; Schilling et al. 2019; Suthakar & Liberman 2019; Thalmeier et al. 2022). Incorporating these correlations in the estimation procedure helps to further reduce uncertainty in the estimated growth function, and as mentioned in the introduction, may lead to a more trustworthy detector, as the detection method’s output is now more in line with examiner’s expectations who similarly exploit these correlations (consciously, or not) when visually inspecting the ABR waveforms.

While the GP has some clear advantages over existing methods, it also has limitations, which first include not exploiting monotonicity in ABR amplitudes across levels. Although the GP exploits smoothness in PTTa values across levels, it allows PTTa values to decrease with increasing stimulus levels. The monotonicity assumption is generally more restrictive than smoothness assumptions, and therefore potentially also more powerful. By leveraging monotonicity in ABR amplitudes, the space of anticipated growth functions can be further reduced, which helps to reduce uncertainty regarding the true PTTa growth function, leading to more efficient growth function estimation.

On a related note, the GP in the present work is applied to just the largest PTTa amplitude values, which typically represent wave V of the ABR. The remaining ABR peaks and troughs may be smaller, but still hold valuable information, which is currently being discarded by the GP. Moreover, by compressing the ABR to a single PTTa value, the GP is also discarding ABR waveform morphology, which holds an additional monotonicity property, that is, increasing ABR peak and trough latencies with decreasing stimulus levels. Further reductions in test time might be obtained by leveraging this additional monotonicity property, along with the remaining ABR peaks and troughs.

Another aspect to consider includes specifying the stimulus levels that the GP is allowed to test at. In the present work, these ranged from −20 to 70 dB with a 1 dB resolution. However, pilot simulations suggest that a 10 dB resolution is slightly more efficient. A 10 dB resolution would also benefit examiners who visually inspect the averaged waveforms together with the GP, as a 10 dB resolution leads to fewer (but higher SNR) coherent averages.

Although GP’s performance is less susceptible to false-positives than conventional detection methods that utilize null hypothesis significance testing with X-down-Y-up test strategies (Chesnaye et al. 2023), it is still adversely affected by spurious patterns in noise. This is apparent when sampling along the $f\left(x_{L},x_{F}\right)=0$ interval, which, in some cases, resulted in the GP temporarily getting “stuck” along this interval. The issue is exacerbated when the GP has no directional guidance regarding the $f\left(x_{L},x_{F}\right)=0$ locations, which occurs primarily in the early stages of the test, that is, when there is relatively little data available. The active learning rules aim to circumvent this issue by not sampling the $f\left(x_{L},x_{F}\right)=0$ interval. This helps to prevent the GP from temporarily getting stuck, but it does not solve the issue entirely, and in some cases, spurious effects along $f\left(x_{L},x_{F}\right)=0$ may still lead to reduced test accuracies and/or increased test times. In future work, more robust solutions should be explored to further mitigate the effect of data outliers.

Last, the GP approach in the present work demonstrated significant reductions in test time relative to the examiners, but the extent to which these test time reductions generalize to a clinical setting remains questionable. This is first because the BSA guidelines in the present work were developed for sleeping infants, rather than awake adults. The adult data recorded in the present work were relatively noisy, and the BSA criteria for response detection were deemed too strict, particularly when finding an acceptable residual noise to determine the absence of an ABR. Although modifications were introduced to keep test time manageable (see Section Visual Inspection by Examiners), these have not yet been optimized, and may have resulted in a less-than-optimal test time. In addition, the examiners in the present work were instructed to adhere to a strict 10-down-10-up stimulus selection protocol with replicate recordings seen at each level, whereas in practice, clinicians may utilize more efficient stimulus selection protocols and not replicate each level tested. It is also worth pointing out that the 10 dB resolution for the stimulus level may have contributed to a reduced test accuracy for the examiners, and that closer approximations of hearing threshold might be achieved with smaller resolutions. Last, the examiners in the present work were audiology graduates who, although had been trained in the 3:1 approach, had limited experience of ABR hearing threshold estimation, whereas, in practice, the test would be carried out by highly trained professionals. These factors may all have contributed toward an overestimated test time for the examiners, albeit relative to what might be observed in the clinic. Future work would be to compare the method on sleeping infants.

### Fully Automatic Versus Assistive Systems for ABR Hearing Threshold Estimation

One question with objective hearing threshold and audiogram estimation is whether to aim to fully replace clinicians, or whether to assist clinicians with this task. The risk with fully automated systems is that they might fail to detect abnormal test conditions, for example, a problem may occur during data collection that is easily picked up by a clinician, but not by the detection method. Similarly, a fully automated system might confuse stimulus artifacts with real responses. As there is much at stake, especially when testing vulnerable patient groups such as infants with hearing loss, it might be unwise to take clinicians out of the loop altogether, albeit until the performance and trustworthiness of these automated systems have reached a sufficiently high standard. That said, there have also been cases of considerable clinical errors in interpreting waveforms in recent years, leading to significant patient mismanagement (British Academy of Audiology 2021). When also considering variations both within and between examiners, an argument can be made to at least assist examiners with ABR hearing threshold estimation using efficient and accurate objective methods.

## CONCLUSION

GPs with active learning were used for real-time ABR audiogram estimation in a cohort of 22 NH and 9 HI subjects. The GP’s median hearing threshold estimation error was 0 dB HL, demonstrating an unbiased test performance relative to the behavioral hearing thresholds to the same stimuli, whilst also reducing test times per frequency by ~50% relative to visual inspection by examiners. The GP utilizes a Bayesian framework for parameter estimation, which is attractive as it circumvents the need for complex sequential tests for controlling FPRs. The GP additionally uses adaptive stopping criteria for data collection, which helps to bring the examination to a clear outcome, thus facilitating quality control of the examination. Last, the GP might be used to fully automate the procedure, or instead might be used to provide timely visual feedback to clinicians, who have the option to manually override the GP’s decisions. Overall, results suggest that GPs with active learning are promising for next-generation ABR threshold-seeking devices.

## ACKNOWLEDGMENTS

The authors acknowledge Gladys Nijo and Prathyusha Sarika for collecting the subject data. The authors would also like to acknowledge the use of the IRIDIS High Performance Computing Facility and associated support services at the University of Southampton.

## Supplementary Material


