# Supplementary material for: Audiogram Estimation Performance Using Auditory Evoked Potentials and Gaussian Processes
Source: Ear Hear. 2024 Sep 12;46(1):230–41. doi: 10.1097/AUD.0000000000001570 (PMC11637572; doi:10.1097/AUD.0000000000001570)
Supplement: Supplementary file 1 [file aud-46-230-s001.pdf]

Supplemental Digital Content for manuscript "Audiogram estimation performance using Auditory Evoked Potentials and Gaussian Processes"

A1. Peak-to-trough amplitude estimation

This section provides a more detailed description on how the (unbiased) peak-to-trough amplitude (PTTa) values were estimated. As mentioned in the main text, the Auditory Brainstem Response (ABR) has a low signal-to-noise ratio (SNR), and many waveforms (or "epochs") need to be averaged before a response can reliably be detected. Each epoch comprises the samples of voltage amplitudes, measured during the short time intervals following stimulus onset using scalp electrodes. For each stimulus level and frequency tested, the coherent average of the epochs can be computed using:

$$\overline{X}_{L,F}(t) = \frac{1}{N_{L,F}} \sum_{n=1}^{N_{L,F}} x_{n,L,F}(t) \qquad \text{Eq. 1}$$

where $x_{n,L,F}(t)$ denotes the voltage amplitude at time t in the nth epoch recorded at stimulus level L (in dB HL) and stimulus frequency F (in Hz), and $N_{L,F}$ denotes the total number of epochs recorded at the given level and frequency. The coherent average $\overline{X}_{L,F}(t)$ is then analysed to estimate the PTTa.

The PTTa values were estimated using the sliding window approach from Chesnaye et al (2023), which constrains the search interval by assuming that the peak precedes the trough, and that the time interval between peak and trough is less than 8 ms. It begins by defining two adjacent 4 ms windows - one for locating the peak, the other for locating the trough - which are slid across $\overline{X}_{L,F}(t)$ in steps of 2 ms. At each window location, a peak and trough are estimated using:

$$P_i = \max_{t \in \mathbf{A}_i} \overline{X}_{L,F}(t) \qquad \text{Eq. 2}$$

and

$$T_i = \min_{t \in \mathbf{B}_i} \overline{X}_{L,F}(t) \qquad \text{Eq. 3}$$

where $\mathbf{A}_i$ and $\mathbf{B}_i$ contain the time points associated with the windows for locating the peak and trough, respectively, at window location i. Taking the difference between P_i and T_i then gives a peak-to-trough difference, and the final PTTa is given by the largest of these differences, taken across all window locations:

$$a_{L,F} = \max_{i \in \mathbf{I}} (P_i - T_i) \qquad \text{Eq. 4}$$

where **I** contains a set of integers for indexing the window positions. A total of 6 window positions were evaluated, giving **I** = [1, 2, 3, 4, 5, 6]. The starting positions for the windows were furthermore set to [2, 4, 6, 8, 10, 12] ms, e.g. when using $i = 1$, $\mathbf{A_1}$ spans the 2-6 ms interval and $\mathbf{B_1}$ spans the 6-10 ms interval - see also **Figure A1**.

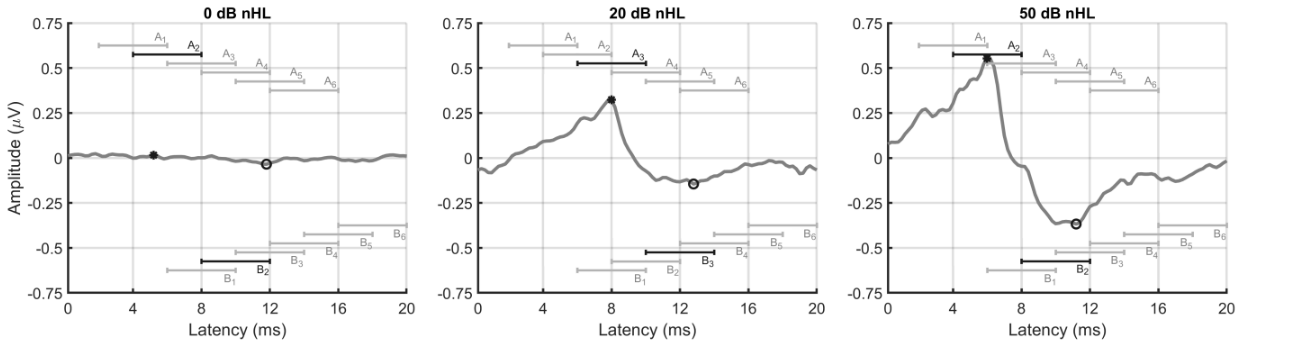

Figure A1. An illustration of the sliding window approach for peak-to-trough amplitude (PTTa) estimation. The approach assumes that the peak precedes the trough, and that the peak and trough are within 8 ms distance of each other. More specifically, two contiguous 4 ms windows are slid across the 2 to 20 ms window of the coherently averaged epoch in steps of 2 ms. At each window location, a peak-to-trough difference is calculated, and the final PTTa is given by the largest of these differences, computed using Eq. 4. **Panel (a)** shows a subject's coherently averaged epoch for the 0 dB Hearing Level (HL) stimulus condition along with all sliding window positions, shown in

light grey. The largest peak-to-tough difference was observed at window position two. **Panels (b)** and **(c)** illustrate the approach for the 20 and 50 dB HL stimulus conditions. This approach was used to estimate the (biased) PTTa values, denoted by $a_{l,f}$, which were transformed to unbiased PTTa values, denoted by $o_{l,f}$, using a maximum likelihood approach.

Unbiased PTTa estimation

An additional complication with PTTa estimation is that the PTTa estimates (the $a_{l,f}$ values) contain a bias due to the residual background activity in $\overline{X}_{L,F}(t)$, which adversely affects the regression analysis conducted by the GP (Chesnaye et al, 2023). It is important to note that although this bias is due to the residual background activity, it is impacted by the ABR's SNR, making it difficult to estimate. This incentivized a maximum likelihood approach for unbiased PTTa estimation.

The maximum likelihood approach aims to replace the biased $a_{l,f}$ estimates with unbiased estimates, denoted by $o_{L,F}$. To do so, it is helpful to first construct the posterior distribution over a range of unbiased PTTa values, conditional on the observed $a_{L,F}$ value. Assuming a uniform prior over unbiased PTTa values, then the (non-normalized) posterior is given by:

$$\Phi_{L,F}(r) = \varphi(a_{L,F} \mid r) \qquad \text{Eq. 5}$$

where $\varphi(a_{L,F} \mid r)$ denotes the expected distribution for the observed (i.e., noisy and biased) PTTa values under the assumption that r is the true (i.e., noise-free and unbiased) PTTa in the data, evaluated at location $a_{L,F}$. The $\varphi(a_{L,F} \mid r)$ distributions can be approximated using a bootstrap approach, as described in Chesnaye et al. (2023). The most likely unbiased PTTa value is then given by the r value that maximizes the posterior:

$$o_{L,F} = \underset{r \in \mathbf{R}}{\arg\max}\ \Phi_{L,F}(r) \qquad \text{Eq. 6}$$

where $\mathbf{R}$ is a vector containing all hypothesized r values.

Finally, the $o_{L,F}$ estimates serve as the data inputs to GP, and need to be delivered along with a level of uncertainty. Each $o_{L,F}$ estimate is therefore also provided with a variance, which is estimated using:

$$\sigma^2{}_{L,F} = \left[\frac{(Q_{0.5} - Q_{0.1587}) + (Q_{0.8413} - Q_{0.5})}{2}\right]^2 \qquad \text{Eq. 7}$$

where $Q_{0.1587}$, $Q_{0.5}$ and $Q_{0.8413}$ denote the r values associated with the 0.1587, 0.5 and 0.8413 quantiles of $\Phi_{L,F}(r)$. Assuming $\Phi_{L,F}(r)$ is approximately normal, then $Q_{0.5}$ is the mean, whereas $Q_{0.1587}$ and $Q_{0.8413}$ are the r values associated with ±1 standard deviation from the mean.
