# Supplementary material for: Audiogram Estimation Performance Using Auditory Evoked Potentials and Gaussian Processes
Source: Ear Hear. 2024 Sep 12;46(1):230–41. doi: 10.1097/AUD.0000000000001570 (PMC11637572; doi:10.1097/AUD.0000000000001570)
Supplement: Supplementary file 2 [file aud-46-230-s002.pdf]

Illustrations of the Matlab Interface for visually inspecting the Auditory Brainstem Response data.

The Matlab Interface used by the examiners to visually inspect the Auditory Brainstem Response (ABR) data is illustrated in the Figures below.

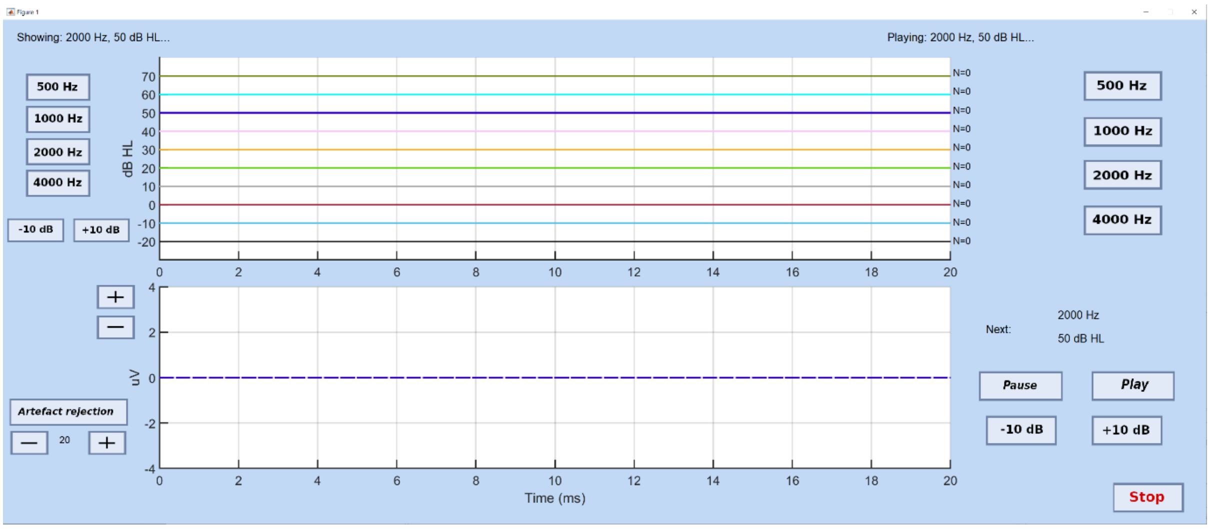

Figure A.1. This figure shows the initial startup screen. There are two main panels that display the averaged waveforms, which are automatically updated (every ~10 seconds, or every 500 stimulus presentations) as data accrues. The top panel shows all coherent averages for a specific stimulus frequency. In this example, the coherent averages are shown for the 2000 Hz chirp for levels -20 to 70 dB HL, in 10 dB steps. As data is yet to be collected, all coherent averages are zero. The number of artefact-free epochs used to compute each average are shown at the right of the waveforms, indicated by "N=0" in the figure.

Moving on to the bottom panel, this shows a zoomed in version of two coherent average replicates for a specific stimulus level and frequency. The latter is selected by the user using the buttons on the top-left, i.e., the user can select the stimulus frequency (using the 500 Hz, 1000 Hz, 2000 Hz and 4000 Hz buttons) and the stimulus level (which can be adjusted in ±10 dB steps). The + and – buttons below are used to zoom in/out along the y-axis, and the + and – buttons at the bottom-left of the panel are used to adjust the artefact rejection level. The latter, however, were not used, i.e., the artefact rejection level remained fixed at 20 µV.

Lastly, there is a set of buttons on the right, which can be used to specify the next stimulus to present to the subject. This includes the stimulus frequency, specified through the 500, 1000 2000 or 4000 Hz buttons, and the stimulus level, which can be adjusted in ±10 dB steps. The interface also shows which stimulus is currently being played (top-right corner), as well as the next stimulus to play (mid-right, above the play and pause buttons), and the stimulus associated with the coherent averages that are currently being displayed (top-left corner). Finally, the user has the option to pause stimulus presentation if needed, e.g., if the subject requests a break.

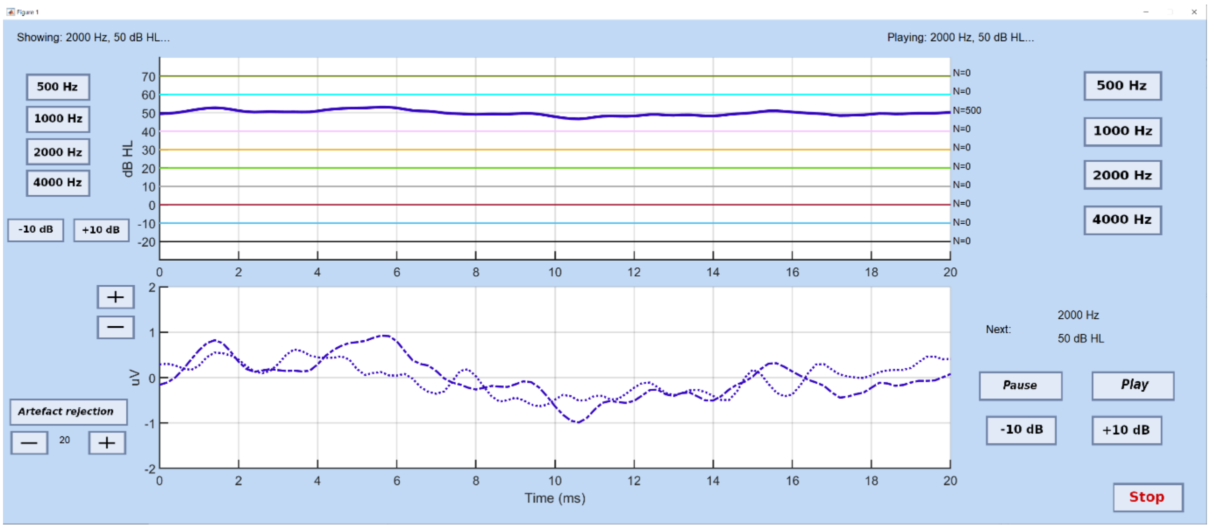

Figure A.2. Here, 500 artefact-free epochs have been recorded for a 2000 Hz 50 dB HL chirp, and the coherent averages have been updated accordingly. The y-axis of the bottom panel has been adjusted to cover the [-2, 2] µV interval to aid visualisation.

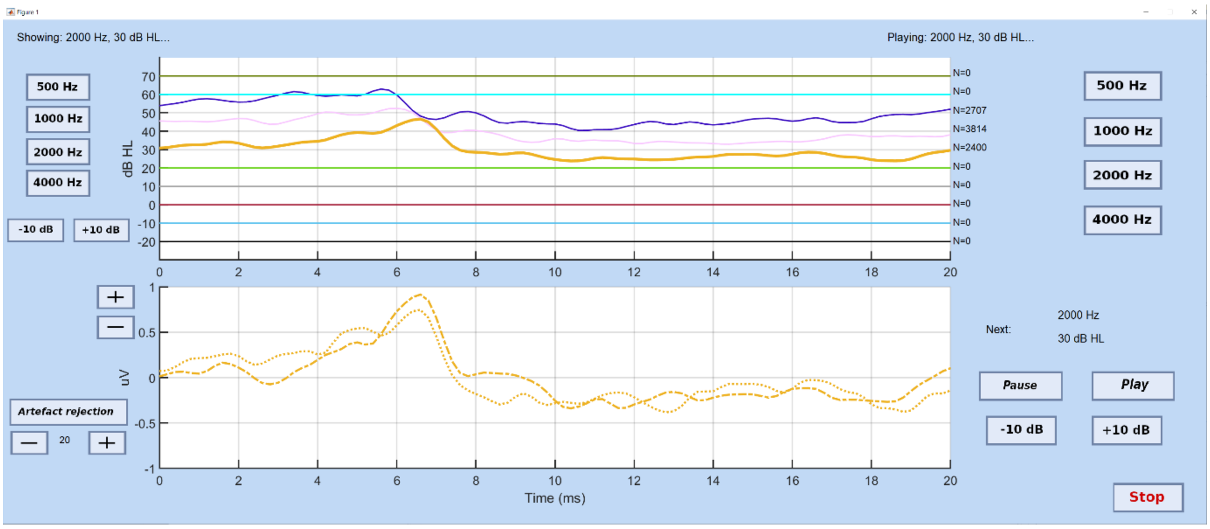

Figure A.3. This figure shows a snapshot of the interface after recording 2707, 3814 and 2400 artefact-free epochs for a 2000 Hz chirp at levels 50, 40 and 30 dB HL, respectively. The y-axis of the bottom panel has been adjusted to cover the [-1, 1] µV interval.

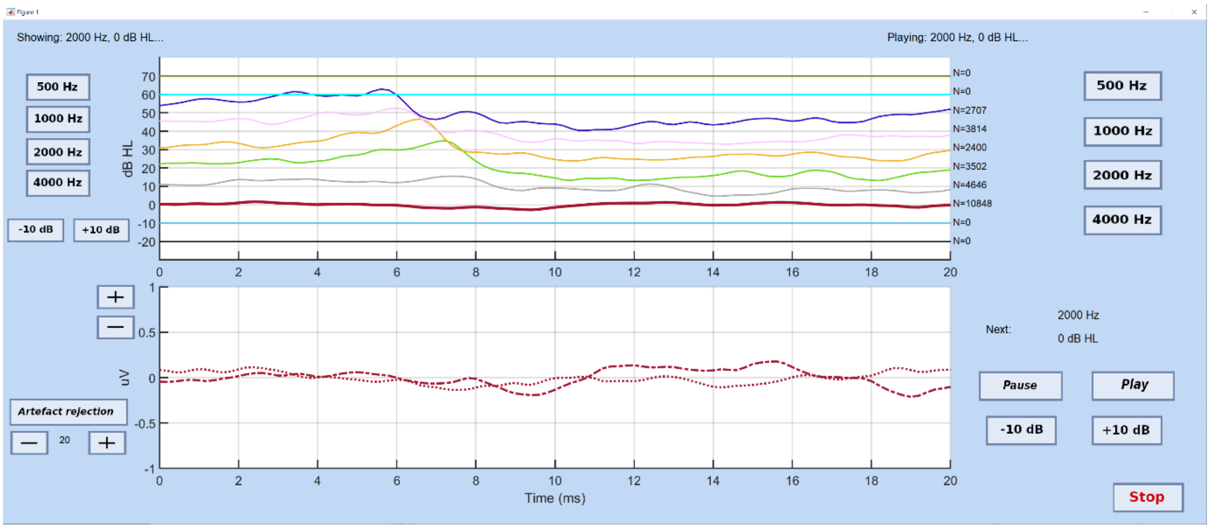

Figure A.4. This figure shows a snapshot of the interface after hearing threshold for the 2000 Hz chirp was determined. In this case, a clear response was deemed present at stimulus levels 50, 40, 30, 20, and 10 dB HL, and a response was deemed absent at 0 dB HL.
